# Gastroprotective Effects of Sulphated Polysaccharides from the Alga *Caulerpa mexicana* Reducing Ethanol-Induced Gastric Damage

**DOI:** 10.3390/ph11010006

**Published:** 2018-01-20

**Authors:** José Gerardo Carneiro, Ticiana de Brito Lima Holanda, Ana Luíza Gomes Quinderé, Annyta Fernandes Frota, Vitória Virgínia Magalhães Soares, Rayane Siqueira de Sousa, Manuela Araújo Carneiro, Dainesy Santos Martins, Antoniella Souza Gomes Duarte, Norma Maria Barros Benevides

**Affiliations:** 1 Department of Biochemistry and Molecular Biology, Federal University of Ceará, s/n Humberto Monte Avenue, Pici Campus, 60455-760 Fortaleza, Brazil; gerardo@ifce.edu.br (J.G.C.); ticipesca@yahoo.com.br (T.d.B.L.H.); aninhaquindere@gmail.com (A.L.G.Q.); annyta.frota@gmail.com (A.F.F.); vitoriabio8@gmail.com (V.V.M.S.); gerardoifce@hotmail.com (R.S.d.S.); manuela.carneiro@hotmail.com (M.A.C.); 2 Federal Institute of Education, Science and Technology of Ceará, Armando Sales Louzada Street, 62580-000 Acaraú, Brazil; 3 Department of Morphoology, Faculty of Medicine, Federal University of Ceará, s/n Delmiro de Farias Street, Porangabuçu Campus, 60416-030 Fortaleza, Brazil; dainy.santos@gmail.com (D.S.M.); asouzagomes@yahoo.com.br (A.S.G.D.)

**Keywords:** marine alga, gastric ulcer, gastroprotection, cytoprotection

## Abstract

The development of the gastric lesion is complex and the result of the imbalance between aggressive and protective factors, involving the generation of free radicals and disturbance in nitric oxide (NO) production. Sulphated polysaccharides (SP), from marine algae, are widely used in biotechnological and pharmaceutical areas. In this study, we evaluated the effects of SP from the green marine alga *Caulerpa mexicana* (Cm-SP) in ethanol-induced gastric damage models in mice. Cm-SP (2, 20, or 200 mg/kg), administered p.o., significantly reduced gastric damage, and these effects were inhibited through pretreatment with indomethacin. Cm-SP (200 mg/kg) prevented the ethanol-induced decline in glutathione and restored its normal level. Moreover, it was able to normalize the elevated thiobarbituric acid reactive substance levels. However, Cm-SP did not show any significant effects on NO_2_/NO_3_ level, when compared to the ethanol group. The pretreatment with L- NAME induced gastric mucosal damage and did not inhibit the gastroprotective effect of Cm-SP (200 mg/kg). In conclusion, the gastroprotective effects of Cm-SP in mice involve prostaglandins and reduction in the oxidative stress and are independent of NO.

## 1. Introduction

Gastrointestinal diseases are an important public health problem affecting many people worldwide. Stress, nutritional disorders, alcohol consumption, prolonged use of non-steroidal anti-inflammatory drugs (NSAID), and glucocorticoids are followed by gastric complications, including stomach ulcers [1,2,3,4]. Their development is complex and the result of the imbalance between aggressive and protective factors, involving the generation of free radicals and disturbance in nitric oxide (NO) production [5,6,7]. In the model of ethanol-induced gastric damage, the administration of absolute ethanol caused macroscopic and microscopic mucosa gastric damage, which is characterized by intense hemorrhage, necrosis, mucosal oedema, and inflammatory infiltrate. Therefore, it is widely used to study gastroprotective effects of new bioactive compounds [1,6]. 

The discovery of new bioactive compounds with potential pharmaceutical activity, presenting minimal adverse effects, is of great importance. Thus, study groups are currently researching new natural compounds and their wide range of applications. Seaweeds are sources of sulphated polysaccharides, and the chemical structure of these polymers varies according to the species [8,9,10]. Sulfated polysaccharides (SP) comprise a group of heterogeneous macromolecules (as alginates, agar, agarose, carrageenans, fucoidan, laminaran, ulvan) presenting various biological effects, with biotechnical potential, and already in use in the food, cosmetics, and pharmaceutical industries [8,9,10,11]. Our group has demonstrated various biological activities of SP, such as anticoagulant [12], antithrombotic [13], neuroprotective [14], antinociceptive, and anti-inflammatory [15,16,17,18].

*Caulerpa mexicana* Sonder ex Kützing is a marine green alga of the Cauleparceae family, which is widely encountered along the coast of Brazil. SP obtained from this alga species have presented antinociceptive and anti-inflammatory effects in a model of ulcerative colitis [19], in oedema caused by histamine and neutrophils migration [18]. However, no study has described the effects of SP from *C. mexicanain* in gastric lesions models. In the present study, we investigated the effects of SP from the green seaweed *C. mexicana* on ethanol-induced gastric damage in mice and the involvement of endogenous prostaglandins (PG) and NO pathway.

## 2. Results

### 2.1. Effect of Cm-SP on Ethanol-Induced Gastric Damage

**Figure 1:**
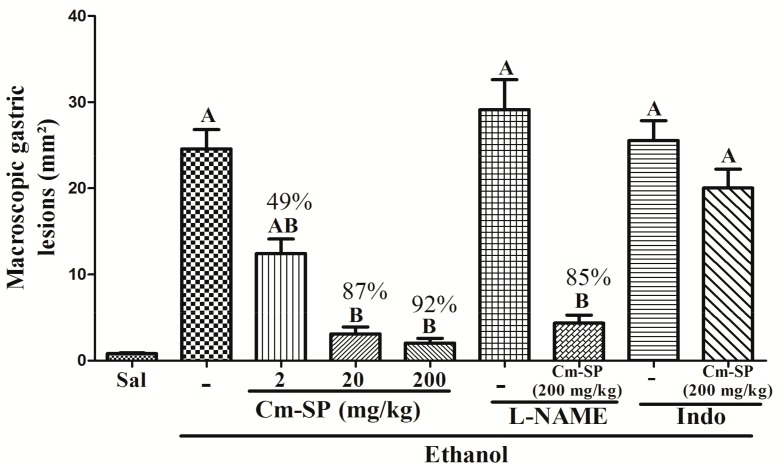
Effect of *Caulerpa mexicana* (Cm-SP) on ethanol-induced gastric damage. Mice were treated with Cm-SP (2, 20, or 200 mg/kg) or saline (sal) by gavage. Another groups received L-NAME (20 mg/kg) or indomethacin (10 mg/kg) (indo). After 1 h, groups were treated with saline (sal) or Cm-SP (200 mg/kg). After 1 h, the gastric damage was induced by administration of ethanol absolute (0.2 mL, p.o.). After 1 h, mice were sacrificed and the total area of macroscopic gastric lesions was determined. The results are expressed as mean ± SEM of a minimum of 6 animals per group. (**A**) *p* < 0.05 vs. saline group; (**B**) *p* < 0.05 vs. group ethanol; ANOVA and Newman-Keuls test.

Oral administration of ethanol absolute (0.2 mL) induced gastric mucosal damage in mice (24.57 ± 2.23 mm^2^). Administration of Cm-SP (2, 20 or 200 mg/kg, p.o.) reduced, in a dose-dependent manner, the gastric damage by 49% (12.41 ± 1.71 mm^2^), 87% (3.09 ± 0.82 mm^2^) and 92% (2.03 ± 0.54 mm^2^), respectively, when compared with the ethanol group (*p* < 0.05). Moreover, the groups that received Cm-SP (20 and 200 mg/kg) did not show any significant difference when compared to the control group saline. The treatment with indomethacin reversed the gastroprotective effects that are promoted by Cm-SP (200 mg/kg). Furthermore, the pretreatment with L-NAME induced gastric mucosal damage (29.12 ± 3.48) and did not inhibit the gastroprotective effect of Cm-SP (200 mg/kg; 85%; 4.34 ± 0.93) (Figure 1).

### 2.2. Histological Assessment

**Figure 2:**
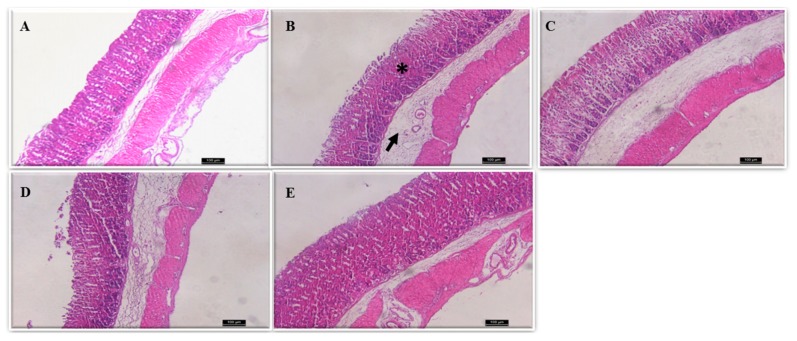
Histological evaluation of the ethanol-induced gastric damage in mice. (**A**) Control stomach: intact gastric epithelium with organized glandular structure and normal submucosa could be seen; (**B**–**E**) ethanol-induced damage; (**B**) mice pre-treated with vehicle: * indicates damaged mucosal epithelium with disrupted glandular structure and arrow depicts oedema of submucosa and inflammatory infiltrate of mucosa; (**C**) Cm-SP 2 mg/kg; (**D**) Cm-SP 20 mg/kg; (**E**) Cm-SP 200 mg/kg. (**C**–**E**) depict a recovery in mucosa epithelium and reorganized glandular structure, as well as improvement of oedema by Cm-SP. (H&E staining; magnification 100×).

Administration of ethanol causes mucosal epithelium damage with disrupted glandular structure, oedema of submucosa, and excessive inflammatory infiltrate (Figure 2). 

However, the administration of Cm-SP (2, 20, and 200 mg/kg) maintained the integrity of the mucosa with uninjured epithelium and organized glandular structure, as well as an improvement of oedema of submucosa and of inflammatory infiltrate, suggesting that Cm-SP exerts a gastroprotective effect. Histological analyses showed that Cm-SP (200 mg/kg) decreased hemorrhagic lesion, oedema, and erosion (loss of epithelial cell architecture), induced by ethanol (Table 1).

### 2.3. Effect of Cm-SP on Malondialdehyde (MDA), Glutathione (GSH), and NO_2_/NO_3_ Levels

Administration of ethanol decreased GSH levels (20.96 ± 1.87), and elevated lipid peroxidation (TBARS content) (391.42 ± 23.06) in gastric tissues when compared to the saline group. Cm-SP (200 mg/kg) prevented the ethanol-induced decline in GSH content and restored its normal level (38.26 ± 0.57). Moreover, Cm-SP (200 mg/kg) normalized the elevated TBARS levels (153.3 ± 8.28). However, the administration of Cm-SP (200 mg/kg) did not show any significant effects on NO_2_/NO_3_ level, as compared to the ethanol group (Table 2). 

## 3. Discussion

The gastric mucosa is continuously exposed to noxious agents and the maintenance of its integrity is ensured by a complex defense system, involving mucus and bicarbonate secretions, modulation of pH, gastric microcirculation, antioxidant factors, PG generation, NO and H_2_S release, and HO-1 pathway induction [5,6,20,21,22]. The pathogenesis of ethanol-induced gastric damage involves direct necrotizing action, mitigation of defensive factors, such as the secretion of bicarbonate and production of mucus, depletion of antioxidant defense, increased oxidative stress, such as free radicals formation and lipid peroxidation, and changes in permeability of mitochondrial membrane prior the increased cell death by apoptosis [1,6,23].

Studies have shown that SP that is extracted from seaweeds exhibit a potential therapeutic effect for several diseases, mainly gastrointestinal tract disorders [9,11,24]. However, studies correlating the gastroprotective action of this polysaccharide are scarce in the literature. Recently, our research group showed that SP enzymatically extracted from the green alga *C. mexicana*, characterized as a mixture rich in sulphated galactan, have presented interesting anti-inflammatory actions with involvement the HO-1 pathway, reduced oedema that is caused by histamine, and inhibited inflammatory cell infiltrate [18]. 

The administration of absolute ethanol caused macroscopic and microscopic mucosa gastric damage characterized by intense hemorrhage, loss of epithelial structure, oedema of submucosa, and inflammatory infiltrate. The results of the present study showed that Cm-SP (20 and 200 mg/kg, p.o.) presented gastroprotective activities, reducing the gastric damage in 87% and 92% of Cm-SP, respectively, when compared with the ethanol group (*p* < 0.05). Furthermore, there was the preservation the integrity of the structure of the epithelium, reduction of oedema of submucosa, and the inhibition of inflammatory cell infiltrate, as verified by histopathological analysis. This data corroborate previous studies on the gastroprotective effects of other SP extracted from marine algae in experimental models of ethanol-induced gastric damage [25,26,27,28].

Several studies show that PG play important roles in modulating the gastric mucosal integrity, mainly against the damaging actions of ethanol. PG are produced from the arachidonic acid by two isozymes of COX, and are main mediators of the inflammatory process [4,7,29]. PG have been implicated in the regulation of various functions of defense gastric mucosal after barrier disruption, including decreased acid secretion, mucus production, mucin release, increased bicarbonate secretion, and mucosal blood flow [4,5,7,22,29,30]. NO is another important mediator of gastric mucosal integrity maintenance. NO’s role has been extensively studied, especially by the use of chemical inhibitors, such as L-NAME. A variety of substances dependent on the NO pathway possess cytoprotective actions [21,31]. Therefore, the inhibition of NO synthesis exacerbates gastric mucosal damage induced by ethanol [21]. However, several studies have shown the gastroprotective action of substances without the involvement of the NO pathway [20,32].

To elucidate the possible mechanism of the gastroprotective action of Cm-SP, we investigated the involvement of PG and NO pathway in the ethanol-induced gastric damage in mice. Our results showed that treatment with indomethacin, a COX inhibitor, reversed the gastroprotective effects promoted by Cm-SP (200 mg/kg), indicating the involvement of PG in the Cm-SP protective effects, showed for the first time that endogenous PG can mediate the SP of seaweeds protection against ethanol-induced damage gastric. Which corroborates with the observations reported in other studies on the gastroprotective action in models of gastric injury [33]. Additionally, the administration of L-NAME, a non-selective inhibitor of NOS, did not inhibit the gastroprotective effect of Cm-SP (200 mg/kg). Since the response to Cm-SP was not affected by L-NAME, it is assumed that the gastroprotective effect of SP is independent of the NO pathway. Therefore, Cm-SP is the first marine algal SP that is reported to present gastroprotective effect independent of the NO pathway.

Several mechanisms are involved in the cytoprotection of the gastric mucosa against damage caused by oxidative stress, including vasoactive metabolites, gaseous mediators (H_2_S, NO, CO), proteins (GSH, HO-1), and appetite peptides (ghrelin, obestatin) [20,32,34,35,36]. Oxidative stress is involved in the pathogenesis of ethanol-induced gastric damage through the formation of free radicals and lipid peroxidation, which is demonstrated by the decrease of GSH and increase of MDA levels in stomach tissue [6,34]. We analyzed two important markers of oxidative stress: GSH, a tripeptide that presents great affinity to H_2_O_2_ and acts mainly as an endogenous antioxidant, being consumed during oxidative metabolism; and MDA, a metabolite of lipid peroxidation. Our findings show that the administration of ethanol induced the generation of free radicals and of lipid peroxidation and that gastroprotective effects of Cm-SP were accompanied by a decrease in oxidative stress in the gastric mucosal, namely, decreased MDA and increased GSH. Our results are consistent with previous reports that demonstrate the involvement of decreased oxidative stress in the action gastroprotective of several SP [25,26,28].

In conclusion, our results indicate that Cm-SP have a gastroprotective activity against ethanol-induced gastric damage, through mechanisms mediated by prostaglandins, independent of the NO pathway and oxidative stress reducer. Thus, we suggest that Cm-SP exhibit potential applications in the development of novel drugs against gastric injury and that further study is needed to explain the mechanism of gastroprotection of Cm-SP.

## 4. Materials and Methods

### 4.1. Animals

Mice (35–40 g) from the Animal Care Unit of the Federal University of Ceará, Fortaleza, Brazil, were used throughout the experiments. For each experiment, groups of six animals were segregated and handled separately. This study was conducted in accordance with the guidelines set forth by the U.S. Department of Health and Human Services, and with the approval of the Ethics Committee of the Federal University of Ceará, Fortaleza, Brazil (CEPA nº. 60/13).

### 4.2. Marine Alga and Extraction of SP

Specimens of *C. mexicana* were collected at Flecheiras Beach, Brazil. A voucher specimen (nº. 47304) was deposited in the Herbarium Prisco Bezerra (EAC) in the Department of Biology, Federal University of Ceará. The SP from *C. mexicana* (Cm-SP) were extracted by the enzymatic method, according to Farias et al. [37], as mentioned in our earlier report [18].

Essentially, 5 g of the dehydrated algal at 25 °C was macerated and subjected to digestion with papain solution (30 mg/mL) in 100 mM sodium acetate buffer (pH 5.0) containing 5 mM cysteine and 5 mM EDTA at 60 °C for 6 h. After, material was centrifuged (2295× *g*, 30 min, 10 °C), the SP in solution were precipitated with 16 mL of 10% cetylpyridinium chloride (CPC) solution. After 24 h at room temperature, the mixture was centrifuged at 2560× *g* for 20 min at 5 °C. The SP in the pellet were washed with 200 mL of 0.05% CPC solution, dissolved with 100 mL of a 2 M NaCl-ethanol (100:15, *v*/*v*) mixture, and precipitated with 100 mL of absolute ethanol. After 24 h at 4 °C, the precipitate was collected by centrifugation (2560 g for 20 min at 5 °C), washed twice with 100 mL of 80% ethanol, and washed once with 100 mL of absolute ethanol. The final precipitate was dialyzed, and freeze-dried. After these procedures, the total SP from *C. mexicana* were obtained [18].

### 4.3. Effect of Cm-SP on Ethanol-Induced Gastric Damage

This assay was performed as described by Damasceno et al. [25], with modifications. Groups of the mice (*n* = 6) were treated with Cm-SP (2, 20, or 200 mg/kg) or saline (0.2 mL) by gavage. After 1 h, the gastric damage was induced by administration of ethanol absolute (0.2 mL, p.o.). To evaluate the involvement of endogenous PG and NO pathway in the Cm-SP action, groups were pretreated with indomethacin (10 mg/kg, p.o.), a cyclooxygenase (COX) inhibitor, or *N*-nitro-l-arginine methyl ester (L-NAME 20 mg/kg, i.p.), a non-selective inhibitor of nitric oxide synthase (NOS). After 1 h, animals were treated, by gavage, with saline (0.2 mL) or Cm-SP (200 mg/kg), and 1 h after the treatment gastric damage was induced by the administration of ethanol absolute (0.2 mL, p.o.). All of the mice were euthanized 1 h after the administration ethanol, and their stomachs were immediately removed and opened via an incision along the greater curvature.

Gastric damage (hemorrhagic or ulcerative lesions) was measured using a computer planimetry program (ImageJ, National Institute of Health, Bethesda, MD, USA). A sample of the corpus region of each stomach was fixed in 10% formalin immediately after removal for subsequent histological assessment. Further, gastric corpus samples were also weighed, frozen, and stored at −80 °C until they were used to determine glutathione (GSH), NO, and malondialdehyde (MDA) levels.

### 4.4. Histological Assessment

Fixed samples of the stomach were dehydrated by increasing concentrations of ethanol and processed for inclusion in paraffin, sliced in 5-μm-thick sections, stained with hematoxylin–eosin (H&E), and then examined under a light microscope by an experienced pathologist. Specimens were evaluated according to Laine and Weinstein [38]. In summary, a 1-cm length of each histological section was assessed for hemorrhagic lesion (a score of 0 to 4), oedema of submucosa (a score of 0 to 4), erosion with loss of epithelial cell architecture (a score of 0 to 3), and presence of inflammatory cells (a score of 0 to 3), with 14 being the maximum score.

### 4.5. Determination of GSH Level

GSH levels in the gastric tissue were measured using the method described by Sedlak and Lindsay [39]. Part of the stomach was used to prepare 10% homogenates (*w*/*v*). Then, the samples were precipitated with 50% trichloroethanoic acid and were centrifuged at 1000× *g* for 5 min. The reaction mixture contained 0.5 mL of supernatant, 2.0 mL of Tris-EDTA buffer (0.2 M, pH 8.9), and 0.1 mL of 10 mM dithiobis (2-nitrobenzoic acid). The solution was kept at room temperature for 5 min, and the absorbance was then read at 412 nm. The results were expressed as micrograms of GSH per gram of tissue (µg/g).

### 4.6. Determination of MDA Level

Lipid peroxidation was estimated by the measurement of the concentration of MDA in the homogenate from each gastric sample according to Draper and Hadley [40], which is based on a reaction with thiobarbituric acid. Samples were mixed with 1 mL of 10% trichloroacetic acid and 1 mL of 0.6% thiobarbituric acid. The reaction medium was heated in a boiling water bath for 15 min, and then n-butanol (2:1 *v*/*v*) was added. After centrifugation (800× *g*, 5 min), thiobarbituric acid reactive substances (TBARS) contents were determined at 535 nm. MDA concentrations were expressed as micromoles per gram of tissue (µmol/g).

### 4.7. Determination of NO_2_/NO_3_ Level

NO level in the gastric mucosa was evaluated as total NO_2_/NO_3_ levels, as described by Souza [14]. Part of the stomach was used to prepare 10% homogenates (*w*/*v*). After centrifugation (800× *g*, 10 min), supernatants were collected, and the NO_2_/NO_3_ production was determined by the Griess reaction [41]. Briefly, 100 μL of the supernatant was incubated with 100 μL of the Griess reagent [1% sulphanilamide in 1% H_3_PO_4_/0.1% *N*-(1-naphthyl)-ethylenediaminedihydrochloride/1% H_3_PO_4_/distilled water (1:1:1:1)] at room temperature for 10 min. The absorbance was measured at 550 nm. The NO_2_/NO_3_ concentration (μM) was determined using NaNO_2_/NO_3_ as standard. The results were expressed as micromols of NO_2_/NO_3_ per gram of tissue (µmol/g).

### 4.8. Statistical Analyses

All data were analyzed using the program GraphPad Prism 7 (GraphPad Software Inc., La Jolla, CA, USA). Differences between means were determined by One-Way Analysis of Variance (ANOVA) and Newman-Keuls test or Kruskal-Wallis test, when appropriate. All data were presented as the means ± standard errors (SEM). Values of *p* < 0.05 were considered to be statistically significant.

## Figures and Tables

**Table 1:** Protective effect of Cm-SP in ethanol-induced microscopic gastric damage.

Experimental Group (*N* = 6)	Hemorrhagic Lesion (Score 0–4)	Oedema (Score 0–4)	Erosion (Loss of Cell Architecture) (Score 0–3)	Cell Infiltrate (Score 0–3)	Total (Scores 14)
Saline	0	0	0	0	0
Ethanol	4 (3–4) ^A^	2 (2–4) ^A^	2.5 (2–3) ^A^	2 (2–3) ^A^	10.5 (9–14) ^A^
Cm-SP (2 mg/kg)	1 (0–1) ^B^	2 (0–3)	1 (0–2)	0 (0–1) ^B^	4 (0–7)
Cm-SP (20 mg/kg)	0 (0–1) ^B^	0 (0–3)	0 (0–3)	0 (0–2)	0 (0–9)
Cm-SP (200 kg/mg)	0 (0–0) ^B^	0 (0–2) ^B^	0 (0–1) ^B^	0 (0–2)	0 (0–5) ^B^

Values denote median with minimum and maximum, respectively. Test of Kruskal-Wallis. ^A^ *p* < 0.05 vs. group Saline; ^B^ *p* < 0.05 vs. group Ethanol.

**Table 2:** Effect of Cm-SP on Malondialdehyde (MDA), Glutathione (GSH), and NO_2_/NO_3_ levels.

Experimental Groups	MDA	GSH	NO_2_/NO_3_
Saline	152.30 ± 9.91	31.81 ± 8.48	16.05 ± 3.24
Ethanol	391.42 ± 23.06 ^A^	20.96 ± 1.87 ^A^	28.02 ± 2.74 ^A^
Cm-SP (200 mg/kg)	153.30 ± 8.28 ^B^	38.26 ± 0.57 ^B^	23.14 ± 3.52
L-NAME	541.60 ± 49.02	19.28 ± 3.74	7.52 ± 1.68
L-NAME + Cm-SP (200 mg/kg)	383.0 ± 23.97 ^C^	32.35 ± 1.56 ^C^	8.66 ± 1.62

Data are expressed as the mean ± SEM (*n* = 6). ANOVA and Newman-Keuls test. ^A^ *p* < 0.05 vs. group Saline; ^B^ *p* < 0.05 vs. group Ethanol; ^C^ *p* < 0.05 vs. group L-NAME.
